# Treatment of Pyorrhea Alveolaris

**Published:** 1902-05-15

**Authors:** Robert Good


### TREATMENT OF PYORRHEA ALVEOLARIS.

BY ROBERT GOOD, D.D.S.

*Mr. President and Gentlemen:*—I have been asked to tell you this evening how I treat pyorrhea. My method of procedure I will try to make plain. I first anesthetize the parts with cocain, ten to fifteen percent, using Parke, Davis & Company's two-and-one-quarter grain tablets, dissolved in a menstruum consisting of the following ingredients:

Fl. opii deod., min. 96;
Normal liq. aconite, gr. 3;
Sat. sol. boric acid, dr. 2.
Sig.—Menstruum for anesthesia.

I make my cocain solution fresh every day, using from fifteen to twenty-five drops of the menstruum to one of the cocain tablets. Sharp & Smith, of Chicago, manufacture a needle especially designed for this work, which I will show to you. I pass the needle to the bottom of the pocket, withdrawing slightly to avoid pressure on the inflamed tissues, which will cause pain. After applying the cocain, wait from one to three minutes before beginning to operate. I use a set of instruments especially designed for this work, which you may see. They are made by Lukens & Whitington, of Philadelphia. The sealing-wax on the handles assists materially in controlling the instrument, and also aids the sense of touch. Using the wax in different colors designates the instrument. I always keep them, while operating, in a solution of sulfo-naphthol.

As the treatment of pyorrhea is purely surgical, it is best to have but one tooth under treatment at a time, finishing it before operating further. After thoroughly removing the deposit, don't allow it to remain in the pocket. Apply chemically pure lactic acid warmed, and be sure to have it reach all parts of the pocket. For this also use a hypodermic syringe, the same as for the cocain. When the packing gives out, have it replaced with a new one. To protect the tissues from the overflow of the acid, pack cotton or bibulous paper around the teeth, and for further protection to the mucous membrane, apply oleostearate of zinc. In cases where the teeth are loose they should be held in a rigid position with bands or silk ligature (No. B sewing silk), splints, or other means. For banding use very thin platinum.

It is essential that every surgical precaution be taken.

Instruct your patients to be particular about keeping the mouth clean. Have them use some good antiseptic mouth-wash. I give mine the following:

ANTISEPTIC MOUTH-WASH.

Thymol, gr. 4;
Benzoic acid, gr. 14;
Tr. Eucalyptus, dr. 4;
Ess. peppermint, dr. 1;
Chloroform, gtt. 15;
Alcohol, oz. 3.

Sig.—Gargle. Twenty drops in glass of water used at a time.

Massage is an excellent thing for the gums, when the soreness leaves, using a little powdered sulphur on the fingers. If a tooth is extremely sensitive it is best to destroy the pulp, because it is an irritant and will prevent a union between the hard and soft parts. In many cases dead pulps will be found; treat them in the usual way. If at the end of three or four weeks a pocket does not close, it is quite certain that a deposit remains; in that event operate again. The successful treatment of pyorrhea depends entirely on the ability of the operator to do thorough surgery. Dr. Harlan has said, "The operator who is able to remove deposit from the roots of teeth is doing the finest surgical work possible."—*Cosmos*.

